# A Case of Circumscribed Dropsy

**Published:** 1891-10

**Authors:** D. B. McGee

**Affiliations:** Brownwood


For Daniel's Texas Medical Journal.

### CIRCUMSCRIBED DROPSY.

BY D. B. M'GEE, M. D., BROWNWOOD.

IF YOU will be kind enough to indulge me space in your valuable journal, I will give the notes of a very peculiar case occurring in my practice a short time since.

Case: E. B., age 9 years, while running across the sleepers of a house before the floor was laid, fell, and sustained an injury in the left hypochondriac region. In about half an hour after passed one pint of bloody urine. Careful examination revealed no broken bones, but some slight bruises, with tenderness on pressure. Hot applications and anodynes relieved some. Heard nothing more from the little fellow until about two weeks afterward, when my attention was called to a tumor developing in the left hypochondriac region. Aspiration proved the contents aqueous. Passed trocar, and drew about three pints of clear water. Sack refilled in about four days, and produced such tension and pressure that the little fellow had some fifteen or twenty reflex convulsions, from which he passed into a comatose state. I again tapped him, relieving him of about half a gallon of water. Reconstructives, generous diet and out door exercise. Refilled again in about three weeks. Same treatment continued. Again tapped him, this time relieving him of about one quart of bloody water. Six weeks have now elapsed since last tapping, and no recurrence of tumor. Now, was not this a case of circumscribed peritoneal dropsy, due to injury to peritoneum from direct violence? Would be pleased to have the opinion of some of the brethren through the columns of the "Red Back." Could not do without the JOURNAL; it is a welcome visitor to my office table.

